# What Makes Employees Green Advocates? Exploring the Effects of Green Human Resource Management

**DOI:** 10.3390/ijerph19031807

**Published:** 2022-02-05

**Authors:** Yufei Cheng, Huanxin Liu, Yiwei Yuan, Zhonghao Zhang, Jinguo Zhao

**Affiliations:** 1 School of Management, Shandong University, Jinan 250100, China; chengyufei_clara@foxmail.com (Y.C.); scholarzzh@126.com (Z.Z.); 2 School of Management, State University of New York at Binghamton, Binghamton, NY 13902, USA; hliu126@binghamton.edu; 3 School of Business, Renmin University of China, Beijing 100872, China; yuanyiwei@ruc.edu.cn; 4 School of Political Science and Law, Qilu University of Technology, Jinan 250353, China

**Keywords:** green human resource management, organization-based self-esteem, employee green advocacy, perceived organizational support, cognitive consistency theory

## Abstract

Green advocacy has been the focus of both practitioners and theorists for decades. However, little attention has been paid to employee green advocacy despite its significance to employee green behaviors and the environmental sustainability of organizations. In an effort to contribute to this nascent field, this study investigates what promotes employee green advocacy and its psychological mechanisms. Based on cognitive consistency theory, we propose that green human resource management (GHRM) can influence employees’ organization-based self-esteem, which motivates them to engage in employee green advocacy to sustain their positive self-image and avoid possible cognitive disorders. Perceived organizational support moderates the relationship between GHRM and employee organization-based self-esteem. Data from a sample of 135 employees and their chief human resource officer (CHO) supported our hypotheses. We discussed the theoretical and practical implications of our findings.

## 1. Introduction

The National Oceanic and Atmospheric Administration reported that the winter of 2020 was the warmest winter dating back to the 1880s [1], leaving people worried about the continuous deterioration of the natural environment [2,3]. To protect the environment, green advocacy has been proposed as an important way to encourage people to behave pro-environmentally [4,5,6,7,8]. Researchers mainly focus on general green advocacy for the public or customers, with little knowledge about employee green advocacy [9,10,11,12,13,14,15,16]. Employee green advocacy refers to the degree to which employees openly discuss environmental issues, communicate various views, and share green knowledge and skills within organizations in order to persuade co-workers to engage in pro-environmental behaviors [17,18]. Studies found that employee green advocacy is beneficial in creating a pro-environmental climate, improving environmental performance, and ultimately achieving organizational sustainable development [17,19,20,21,22], which further highlights the importance for organizations to encourage employee green advocacy [18,23]. However, limited studies have explored the antecedents of employee green advocacy, including leader green behavior [17] and employee perceived corporate social responsibility [24,25]. Green human resource management (GHRM), the human resource management aspects of environmental management [26], was indicated to facilitate green behaviors of employees through influencing their psychological states such as organizational commitment, psychological green climate, and pro-environmental psychological capital [21,23,27,28]. We choose GHRM to further explore its effect on employee green advocacy as well as the underlying psychological mechanism.

In building a model linking GHRM and employee green advocacy, we focus on the literature of GHRM and green advocacy, respectively, to posit a mediating role of organization-based self-esteem (OBSE) and a moderating effect of perceived organizational support (POS). OBSE is defined as the degree to which organizational members believe that they can meet their needs by functioning in the organization [29]. Or in other words, OBSE means the evaluations of ones’ self-value within the organization [30]. According to cognitive consistency theory, individuals tend to engage in behaviors consistent with their self-cognition to maintain a consistent evaluation of self-image and reduce possible cognitive disorders [31]. We posit that GHRM contributes to the OBSE of employees by signaling their high self-value within organizations, and employees with high OBSE tend to take actions to prove and enhance their value. Prior research proposed that whether individuals initiate advocacy depends on their perceived personal accountability [32,33,34,35]. GHRM improves employees’ OBSE, promoting their faith in themselves. Employees are more likely to feel accountable for the environment and encourage others to behave pro-environmentally. Thus, we regard OBSE as the potential psychological mechanism of employee green advocacy.

**Figure 1:**
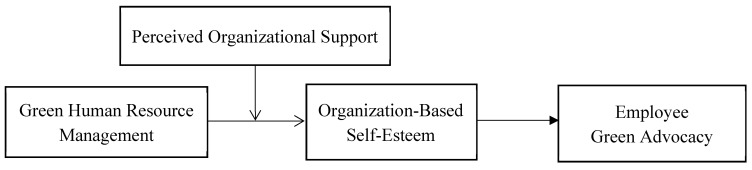
Theoretical model.

The moderating mechanism, POS, is the extent to which individuals believe that their organization values their contributions and cares for their well-being [36,37]. According to cognitive consistency theory, individuals have the tendency of maintaining a consistent understanding of things [31]. When people get consistent information about one specific thing from different sources, they will feel comfortable and reinforce their view towards it; however, when they get inconsistent information, they will be psychologically uncomfortable because of the lack of cognitive consistency and will take measures to diminish the cognitive disorder [38,39]. As both GHRM and POS help employees perceive being valued by the organization, we posit the interaction of GHRM and POS will help employees possess the cognitive consistency that they are valued by the organization and are competent for the job, which could exert an enhanced impact on their OBSE [29]. On the contrary, if employees perceive low POS in the process of GHRM, they will perceive inconsistent messages of their self-worth from GHRM and low POS. The contradictory recognition will cause them cognitive disorder. To mitigate the cognitive dissonance, employees may change their views of being valued and competent, which will consequently weaken their OBSE [29]. As illustrated above, we propose POS functions as a moderator in the relationship between GHRM and OBSE. Our research model is illustrated in Figure 1.

The contributions of our research are threefold: First, this study proposes and tests the psychological mechanism through which employee green advocacy was affected, filling the gap in green advocacy literature. We present a conceptual framework and examine the role of GHRM and employees’ OBSE, contributing to our understanding of antecedents of green advocacy. Second, by investigating the effect of GHRM on employee green advocacy through OBSE, this study responds to the call from Tang and her colleagues [40], which suggests the great necessity of future studies to explore more factors influenced by GHRM and more mediating mechanisms through which GHRM exert influence. Third, this study also enriches the POS literature by examining its moderating role in the relationship between GHRM and OBSE.

## 2. Theoretical Background and Hypotheses Development

### 2.1. Cognitive Consistency Theory

Cognitive consistency theory includes congruity perspective, which indicates that individuals have cognitive consistency toward things [39], and cognitive dissonance perspective, which argues that human may possess cognitive disorders because of conflicting knowledge from different sources [38,41]. An important and unique feature of cognitive consistency theory is its emphasis on individual’s consistency cognition [31]. Individuals’ cognition is literally influenced by mutual interaction of information from different sources [42]. Individuals tend to perform certain behaviors to keep their cognition consistent and avoid potential cognitive disorders [31].

Cognitive consistency theory not only explains the reasons why individuals perform specific behaviors from a psychological level, but also focuses on how individuals’ psychological status and behaviors can be influenced when individuals are exposed to messages from different sources [42]. Using cognitive consistency theory, we can better understand employees’ behaviors within organizations [31]. Employees are exposed to many informational resources that may shape their cognition within organizations. For example, organizational supportive practices can convey the important messages to employees that they are valued and supported by their organizations [43]. In this circumstance, employees will gradually build up front self-cognition. To sustain their positive self-cognition within organizations, employees would like to do something beneficial to the organizations [31]. Employee green advocacy is a kind of green behavior that contributes to improving organizations’ environmental performance [17]. Based on cognitive consistency theory, this study wants to explore the factors that influence employee green advocacy. First, we want to explore the impact of GHRM on employee green advocacy. GHRM practices align organizations’ environmental strategies with employees’ pro-environmental participation [44]. To some extent, GHRM can not only reflect the implementation of environmental strategies but also directly affect employee pro-environmental awareness and action [21]. Thus, information derived from GHRM progress seem to be vital for employees to evaluate themselves [21]. Second, we expect GHRM can positively affect employee green advocacy via their OBSE. OBSE reflects a kind of positive self-cognition of individuals [29]. GHRM practices show the important role organizations place on their employees in enhancing green performance [21]. Employees are more likely to perceive that they are valued by organizations and build up front self-cognition via participating in GHRM practices. To sustain their positive self-cognition within organizations, employees would like to engage in green activities (green advocacy) [17,20,21]. Third, we propose that the interaction of GHRM and POS, as two distinguished information resources, may saliently influence employees’ self-cognition: OBSE. POS, directly representing care from organizations to employees [45], determines employees’ judgment of the organization’s intention to implement GHRM [46]. If the levels of GHRM and POS are both high, their synergy will benefit employees’ positive cognition, their OBSE. However, if the levels of GHRM and POS are different (for example, the level of GHRM is high but the level of POS is low), employees will develop inconsistent cognition of themselves, doubting if they are truly valued by organizations.

Therefore, cognitive consistency theory can sufficiently explain the mechanism of our research model.

### 2.2. Green Human Resource Management and OBSE

Defined as the environmental aspect of management, GHRM aims at improving environmental performance and eventually contributing to environmental sustainability of organizations [44,47]. GHRM includes five major initiatives: green recruitment and selection, green training and development, green performance management, green pay and reward, and green involvement [26,44,47,48,49,50,51]. Presumed to be positively influenced by GHRM in this study, OBSE reflects the evaluation of one’s self-worth in the organization [30]. Based on the literature of OBSE, we know that organizational members with high OBSE are usually the ones who recognize their self-worth and gain trust from other organizational members for their competence as organizational members [52,53,54]. We propose the implementation of GHRM can satisfy employees’ OBSE, the specific psychological needs of employees within organizations. Next, we will discuss the relationship between the five GHRM practices and employees’ OBSE to investigate the relationship between GHRM and OBSE.

#### 2.2.1. Green Recruitment and Selection and OBSE

Green recruitment and selection is defined as the process of seeking appropriate candidates with green values, knowledge, skills, and behaviors instrumental to the environmental management system within the organization [26,55]. Candidates chosen by green recruitment and selection will easily realize that their environmental knowledge and skills will be needed and valued by the organization. As OBSE reflects one’s self-perceived value within organizations [29], we suppose the more employees believe organizations value themselves, the higher OBSE they will possess.

#### 2.2.2. Green Training and Development and OBSE

Green training and development is a process that not only helps employees realize the environmental effect of their organization’s activities [56,57], but also cultivates employees with knowledge and skills related to environmental management [58]. Both providing employees with opportunities to participate in green training and focusing on their green career development will strengthen their sense of being valued by the organization and facilitate the crystallization of their OBSE [29].

#### 2.2.3. Green Performance Management and OBSE

To achieve better green performance, companies utilize a green performance management system to assess environmental performance of employees [59,60]. Green performance management refers to the process in which corporations adopt corporate-wide environmental performance standards to assess employees’ utilization, conservation, and waste of resources [59]. Set in a green performance management system [26,44], green goals and performance indicators represent the high expectations from the organization and managers to employees. As the expectations from others are proved to play a shaping role in individuals’ OBSE [61], a green performance management system positively contributes to the OBSE of employees [61].

In addition, Korman [61] believes that the evaluation from others, especially the evaluation from important persons in the organization, will have a significant impact on an individual’s OBSE. As a part of green performance management [60], leader feedback represents leaders’ evaluations of employees and thus may influence employees’ OBSE markedly.

#### 2.2.4. Green Pay and Reward and OBSE

Green pay and reward is the practice of providing employees with rewards for their efforts and achievement in realizing green goals set in green performance management systems [60,62]. Presented in many forms, such as monetary-based rewards, non-monetary based rewards, recognition-based rewards, and positive rewards [26,44,60], green pay and reward signals that organizations value employees who commit to achieving green goals and contributing to environmental sustainability of the organization [26,60]. With such a hint, green pay and reward will contribute to employees’ OBSE. Moreover, green pay and reward reflects the organizations’ positive evaluations of green efforts of employees, which is also beneficial to the OBSE of employees [29].

#### 2.2.5. Green Involvement and OBSE

As Renwick et al. [26] described, green involvement refers to a process that managers provide opportunities to subordinates to participate in environmental management and address environmental problems emerging in the organization. Through green involvement, employees are more able to raise novel ideas contributing to environmental management and express their own opinions of approval of or disagreement with decisions concerning organizational green issues [19,55]. Therefore, green involvement, in which employees’ ideas and suggestions are respected and may be adapted to a great extent [55], can help employees enhance their OBSE by making them feel that their competence is admitted by the organization [29].

Summarizing the above arguments, we believe that GHRM can positively influence employee OBSE and propose the following hypothesis:

**Hypothesis** **1** **(H1).** *GHRM will be positively associated with employees’ OBSE.*

### 2.3. The Mediating Role of Organization-Based Self-Esteem

Following Kim et al.’s [17] conceptualization, we define the term employee green advocacy as the extent to which employees openly discuss environmental issues, communicate diverse opinions, and share green knowledge within the organization in order to encourage other organization members to engage in pro-environmental behaviors and eventually contribute to organizational environmental sustainability. Different from “eco-helping” that focuses on helping co-workers in environmental aspects, employee green advocacy lays emphasis on persuading and calling on colleagues to engage in environmental behaviors [17,63]. Based on cognitive consistency theory, we propose that the OBSE of employees has a salient effect on their green advocacy. According to cognitive consistency theory, individuals will actively engage in behaviors consistent with their self-cognition to maintain a consistent evaluation of self-image and reduce possible cognitive disorders [31]. In other words, if employees perceive that they are valued by the organization (OBSE), they will commit behaviors beneficial to the organization to prove and strengthen their value.

We posit GHRM practices motivate employees to engage in green advocacy partly through satisfying their emotional needs of OBSE. The possible positive relationship between GHRM and OBSE has been discussed in the previous section. We also suppose employees with strong OBSE, who have already recognized their high self-value within the organization, tend to continue doing things valuable to the organization, to sustain and enhance their value, and to avoid cognitive disorders of themselves [31]. Because employee green advocacy conforms to organization’s green values and is constructive to the sustainable development of the organization [17,64,65], it is reasonable for employees who already perceived their high self-value within organizations (strong OBSE) to prove their value by engaging in green advocacy to sustain their positive self-cognition consistency. Moreover, studies indicate that the reason why individuals initiate advocacy concerns their perceived personal accountability [32,33,34,35]. Employees who believe that they are accountable in specific areas are more likely to be the advocates of this field [32,33]. As illustrated above, GHRM improves employee OBSE, promoting employees’ faith in themselves. Employees who believe in their environmental accountability are more likely to encourage coworkers to behave pro-environmentally and become green advocates within organizations.

As illuminated above, the following hypotheses were formulated:

**Hypothesis** **2** **(H2).** *Employees’ OBSE will be positively associated with their green advocacy.*

**Hypothesis** **3** **(H3).** *Employees’ OBSE plays a mediating role in the relationship between GHRM and employee green advocacy.*

### 2.4. The Moderating Role of Perceived Organizational Support

POS is defined as the extent to which individuals believe that the organization they work for values their contributions and cares for their well-being [36,37,66]. We will discuss the moderating role of POS in the relationship between GHRM and OBSE according to the five dimensions of GHRM in the following section.

We suppose if organizations show care and attention to pro-environmental applicants (high POS) in the green recruitment and selection process, applicants will be more convinced that their green values are consistent with the organizations’ and they are the ones needed by the organizations, thus they are more likely to build up OBSE [29,67,68]. In addition, when employees perceive high POS during their green training and development, on account of social exchange, they will study green knowledge and sharpen green skills more passionately to be more competent for green work than those feeling less support [66,69,70]. Such a sense of competence in their work favors their OBSE enhancement [29].

In the process of green performance management, if organizations set green performance standards in a more humanized way and care about employees’ feelings when sharing appraisal results with them (high POS), such as praising employees with good performance while encouraging those with poor performance, these practices will fulfill employees’ socioemotional needs of being trusted and respected [71], further improve their confidence in themselves, and contribute to their OBSE [29]. Following performance management, when organizations reward employees with what they desire (high POS), employees will be more willing to believe that they are cherished by the organization and will strengthen their OBSE [29]. In the process of green involvement, we posit that if organizations allow employees to be involved in organizational green issues and attach importance to their suggestions (high POS), employees will be more convinced that they are truly trusted by the organization and evaluate their OBSE to a higher level [29].

To summarize, we argue that high POS advances the promoting effect of GHRM on OBSE, whereas low POS weakens the promoting effect of OBSE by GHRM. Cognitive consistency theory indicates that individuals’ cognition towards something tends to be consistent [31]. When individuals get similar cognition of something from different sources, it will strengthen their conclusions towards it. In contrast, if they get inconsistent cognition about something from several sources, they will possess a psychological discomfort and will take measures to narrow cognitive disorders [38,39]. Obviously, GHRM and POS are two different sources for employees to evaluate themselves. We posit that if employees evaluate themselves positively from the sources of both GHRM and POS, they will possess a consistent cognition of themselves. Such cognitive consistency will strengthen their view that they are competent for their work, which will improve their OBSE. However, if employees get a positive evaluation of themselves from the source of GHRM while getting a negative one from the source of POS, they will draw contradictory conclusions about themselves and will have cognitive disorders. An important observation of the cognitive consistency theory is that consistency is regained by changing the elements in the discordant relationship or reducing the importance of discordant elements [38,42]. To reduce the psychological discomfort brought by cognitive disorders and regain cognition consistency, employees may change their mind of thinking highly of themselves and regard the idea that they are competent for work as invalid, which is deleterious to their OBSE [29].

Above all, the theory-driven line of reasoning leads to the derivation of our fourth hypothesis as follows:

**Hypothesis** **4** **(H4).** *POS moderates the relationship between GHRM and OBSE. When employees possess a higher degree of POS, they are more likely to build up stronger OBSE after the implementation of GHRM, compared to employees with a lower degree of POS.*

## 3. Method

### 3.1. Sample and Procedures

It is important to understand the great role of Chinese employees in helping protect the environment. First, corporate environment management has received much attention in China owing to growing public concerns and governmental pressures on environmental protection [72]. Employees’ green awareness and actions can contribute greatly to corporate environmental performance [73]. Thus, it is practical to investigate the factors affecting employees’ green action. Second, the environmental management experience in Chinese corporations can provide references for other countries, especially for developing countries. China is the largest developing and manufacturing country in the world [74]. However, the rapid economic growth has caused great environmental damage due to the massive use of high-pollution natural resources (e.g., fossil fuel and coal). Thus, in order to reduce the environmental threats, Chinese government makes great efforts (e.g., issued the Environment Protection Law in 2014) on reducing the pollution caused by development [75]. Under this circumstance, Chinese corporations put environmental management into practice to pursue sustainable development [72]. Such experience is typical and helpful for corporations in other developing countries that may meet the similar environmental problems. Therefore, we tested our hypotheses in the Chinese context.

Considering the availability of data collection, we invited 145 MBA students from universities in eastern China and their Chief Human Resource Officers (CHO, one employee matched with one CHO) to complete our questionnaires. These MBA students are also employees from 145 different companies in China, and the MBA programs they took are not full-time courses. In other words, they can work as usual but take MBA courses on the weekends. In addition, the majority of participants are less than 40 years old. It is vital to investigate how corporations cultivate employees’ green awareness and actions at a relatively young age. Compared to older employees, younger employees are less likely to prioritize emotionally meaningful social goals; they tend to place greater emphasis on knowledge gathering and career development [76]. Therefore, it is necessary to identify the factors that affect younger employees’ pro-environmental actions (which are also pro-social actions). We collected data at two different times to reduce the homologous errors [77]. We invited CHO to complete questionnaires including GHRM on the Time 1 survey. At Time 2, about 2 months later, employees were invited to assess their OBSE, POS, and green advocacy. They were also required to fill in their background information (demographic information like age, education; other information like organizational tenure, employment type, and organizational size). Participants were informed that the survey was only for academic purposes with strict confidentiality.

After deleting mismatched questionnaires, we had a final sample of 135 sets of paired data with a total response rate of 93%. Table 1 shows the demographic composition of the sample. As shown in Table 1, the majority (88.6%) of the participants were formal workers, and a large proportion (55.5%) of them were within the age range of 30–39 years. Concerning education information of employees, 41.4% were of or above undergraduate education, whereas 58.6% were below the undergraduate education. As for the organizational tenure of employees, 30.5% were above 3 years and under 5 years, and 29.1% were above 5 years and under 10 years. For the organizational size, 39.3% of participants reported that their organizations contained more than 50 employees but fewer than 100 employees.

### 3.2. Measures

To design our own questionnaire items, we followed the literature that has been published. A translation-back procedure was adopted for questionnaires from English literature. The questionnaires were first prepared in English and then translated into Chinese by a professional translation service company according to the back-translation strategy to ensure that the semantics before and after translation remained the same [78]. Before releasing the formal survey, some MBA students were invited to evaluate whether the questionnaires were understandable. After that, we did a minor revision of the wording. Unless otherwise specified, all measures in this study were evaluated by a 5-point Likert-scale ranging from 1 (strongly disagree) to 5 (strongly agree) as described below. More detailed items of constructs of variables of this study are given in Appendix A.

#### 3.2.1. Control Variables

We controlled for employee’s demographic variables like age, education, and other variables like organizational tenure (years employees worked in the organization), organizational size (the number of employees in the organization), and employment type (formal employee or temporary employee). Because employees with longer tenure usually have greater opportunities to engage in social interactions that strengthen their emotional links with co-workers and increase their confidence to engage in green advocacy [24,79], it is advisable to control for the potential influence of the organizational tenure of employees. In addition, formal employees are more committed to the organization they work in than temporary employees [80], so they are more likely to engage in activities that are consistent with organizational value, like green advocacy. Therefore, we added employment type into control variables. Moreover, we controlled for organizational size because it may exert a great impact on employee green advocacy [17,81].

#### 3.2.2. Green Human Resource Management (GHRM)

GHRM was rated by the CHOs using 18 items developed by Tang et al. [44]. The items consist of green recruitment and selection, green training, green performance management, green pay and reward, and green involvement. Cronbach’s alpha was 0.957.

#### 3.2.3. Employee Green Advocacy

Employee green advocacy was rated using the three items developed by Kim et al. [17]. Cronbach’s alpha was 0.922.

#### 3.2.4. Organization-Based Self-Esteem (OBSE)

OBSE was rated on 10 items taken from the scale developed by Pierce et al. [29]. Cronbach’s alpha was 0.971.

#### 3.2.5. Perceived Organizational Support (POS)

POS was measured using six items adapted from the Survey of Perceived Organizational Support (SPOS, Eisenberger et al. [37]). This shortened version of the SPOS has been used in previous research [82,83]. Cronbach’s alpha was 0.808.

### 3.3. Analytic Strategy

Mplus 7.0 was employed for confirmatory factor analysis and SPSS 21.0 was used for correlation analysis. In addition, the SPSS macro process was utilized to test our hypotheses in two interlinked steps: Model 4 was first selected to examine the mediation model (Hypotheses 1, 2, and 3), and then Model 1 was chosen to test the moderated effect (Hypothesis 4). All continuous variables have been centralized before the analyses [84].

### 3.4. The Analytic Method of Mediation

As Sobel [85] test and bootstrapping is the best known among the test methods [86,87], we tested the mediation hypothesis (Hypotheses 1–3) with an SPSS macro process (developed by Preacher and Hayes [87]), which is conducive to the estimation of the indirect effect, through both a normal theory approach and a bootstrap approach to get CIs. Specifically, we chose Model 4, and then we put GHRM into X, OBSE into M, and employee green advocacy into Y. The control variables (employees’ age, education, organizational tenure, employment type, and organizational size) were put into covariates.

### 3.5. The Analytic Method of Moderating Effect

For Hypothesis 4 we predicted that POS would moderate the positive relationship between GHRM and OBSE. We also adopted an SPSS macro, which provided an effective way to probe the significance of the moderating effect under different moderator variable values. Specifically, we chose Model 1 of the process, and then we put GHRM into X, OBSE into Y, and POS into M. The control variables (employees’ age, education, organizational tenure, employment type, and organizational size) were put into covariates.

## 4. Results

### 4.1. Common Method Bias

In order to reduce possible common method bias, we not only collected the measured values of independent and dependent variables from different informants, but also used various scales to measure the variables [77]. We also adopted following methods to test possible common method bias.

First, we used Harman’s one-factor test of common method bias as in previous research [88,89]. All items of the four variables in our study (GHRM, OBSE, employee green advocacy, POS) were analyzed by non-rotating exploratory factor analysis. A total of four factors with characteristic root values greater than 1 were separated out. The first factor explained 38.973% of the total variance variation, which was less than the standard of 40% proposed by Podsakoff and his colleagues [77]. Second, as shown in Table 2, the confirmatory fitting analysis results of single factor models for all items of the four variables does not fit the data (χ^2^/df = 11.770; RMSEA = 0.265; CFI = 0.664; TLI = 0.581; SRMR = 0.164), indicating that there is no common factor extraction for all items of the four variables. The two methods illustrated above both prove that common method bias of this study is not obvious, thus the influence of common method bias will not be considered in this study.

### 4.2. Confirmatory Factor Analysis

Before forming the scales for hypothesis testing, we assessed the construct validity of our measures using confirmatory factor analysis (CFA) by comparing the measurement model with four competing models, described in detail in Table 2 [90]. Confirmatory factor analyses were conducted to establish the distinctiveness of GHRM, employee green advocacy, POS, and OBSE. Three item parcels for the OBSE scale and two item parcels for the POS scale were used, and these parcels were created using a factor loading strategy based on a preliminary analysis at the item level (i.e., having one strong loading, on weak loading, and one or two moderate loading in each parcel) [91]. Moreover, we first used five item parcels for the GHRM scale according to the five dimensions of GHRM [44], given the number of items compared with the study’s sample size, and then we parceled the five parcels into three according to the factor loading strategy noted above to get a better fitting degree. As shown in Table 2, our four-factor measurement model was the best-fitting.

The expected four-factor model (a priori model) was compared with four plausible alternative models, and the CFA results show that the expected four-factor model was the best-fitting model in comparison with the alternative models (see Table 2), which provided a reasonable fit to the data, supporting the unidimensionality of our measures: comparative fit index = 0.993; Tucker–Lewis index = 0.989; root mean square error of approximation 90% confidence interval = 0.000, 0.081.

### 4.3. Measurement Model Assessment

The reliability of the constructs in this study was evaluated by Cronbach’s alpha. All Cronbach’s alpha values were higher than the suggested threshold value of 0.7, which proved that the measurement for all constructs was reliable [92]. The validity of the measurement was examined as follows. First, the measurement items were adapted from previous literature, which contributes to the content validity. Second, as shown in Table 3, all composite reliability values exceed the threshold value of 0.7. In addition, the factor loading values as well as the average variance extracted (AVE) values were larger than the threshold level of 0.5. These findings generally indicate convergent validity [92,93]. Third, Table 3 shows that the square root value of each construct’s AVE was larger than all its correlation coefficients with other constructs, therefore discriminant validity was hence proved to be sufficient in this study [93].

### 4.4. Correlation Analysis

Descriptive statistics, mean, standard deviations, and correlations for all variables in the study are presented in Table 4. As seen in Table 4, GHRM was positively related to employees’ OBSE (b = 0.424, *p* < 0.01), employees’ OBSE was positively related to the flourish of employee green advocacy (b = 0.650, *p* < 0.01), GHRM was positively related to employee green advocacy (b = 0.411, *p* < 0.01), POS was positively related to GHRM (b = 0.406, *p* < 0.01), and POS was positively related to employees’ OBSE (b = 0.571, *p* < 0.01). Such low to moderate correlations suggest that these variables represent different concepts. These results were consistent with the direction of this study and provided preliminary data to confirm our hypotheses.

### 4.5. Tests of Mediation

Table 5 presents the results for hypotheses 1–3. Supporting hypothesis 1, GHRM was positively associated with OBSE, as indicated by a significant unstandardized regression coefficient (B = 0.318, *t* = 5.608, *p* < 0.01). Also, in support of Hypothesis 2, the positive relationship between OBSE and employee green advocacy was supported (B = 0.563, t = 5.644, *p* < 0.01). Eventually, OBSE was found to be a mediator and has an indirect effect on the relationship between GHRM and employee green advocacy. With a bootstrapped 95% CI not including zero [0.093, 0.334], the total effects of GHRM on employee green advocacy in this model was significant (B = 0.213, *p* < 0.01). The results proved the indirect effect was positive (0.095) as we predicted (Hypothesis 3). The indirect effect was significant (Sobel z = 2.916, *p* < 0.01) as shown in the model with the formal two-tailed significance test. The 95% bootstrapping CIs for the indirect effect excluded zero (as shown in Table 5), providing support for Hypothesis 3.

### 4.6. Tests of Moderation

**Figure 2:**
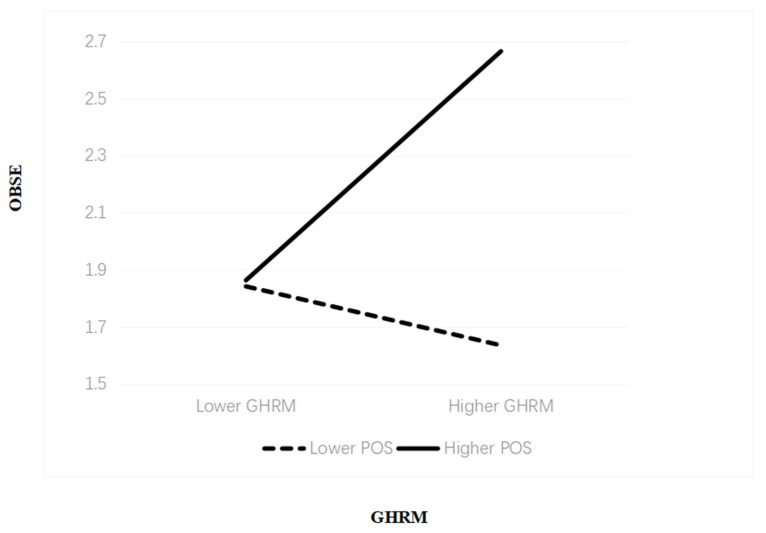
Plot of the interaction effect of GHRM and POS on OBSE.

Hypothesis 4 predicted that POS moderated positively the positive relationship between GHRM and OBSE. As shown in Table 6, the strengthening role POS was statistically significant (B = 0.252, t = 2.319, *p* < 0.05). GHRM had a stronger relationship with OBSE when organizational members are measured to possess a higher (B = 0.247, *p* < 0.01) degree of POS versus the lower (B = 0.149, *p* < 0.01), and the specific description was shown in Figure 2. Therefore, Hypothesis 4 was supported.

## 5. Discussion and Implications

### 5.1. Discussion

Employee green advocacy, characterized by the positive actions organizational members take to encourage others to behave environmentally, is largely ignored by researchers and managers concerning its great contributions to organizational environmental performance [17,94]. In order to gain a better understanding of green advocacy, this study investigated why employees act as green advocates within organizations; namely, that GHRM may promote employees’ OBSE and motivate them to act as green advocates to sustain positive self-value and maintain cognitive consistency about themselves. Our study examined the relationship between GHRM and employee green advocacy. As predicted, GHRM was positively associated with employee green advocacy. Our results showed that OBSE mediated the relationship between GHRM and employee green advocacy. What is also indicated in our results is that POS moderated the relationship between GHRM and OBSE. When POS was high, the positive relation between GHRM and OBSE was strengthened. Specifically, two main conclusions can be reached from this study.

First, OBSE was a reasonable explanatory mechanism for understanding why GHRM predicts employee green advocacy behaviors. This conclusion is in line with the claim of Ren and her colleagues [21] that GHRM could bring about changes in employees’ attitudes and behaviors. The conclusion is also consistent with previous research indicating that GHRM practices facilitated pro-environmental organizational citizenship behaviors of employees [18]. In addition, our study responds to Jackson and Seo [95], who called for more research to understand employee discretionary behaviors that are beneficial for organizations to be greener.

Second, our results suggest that under a high level of POS, the positive influence of GHRM on employees’ OBSE is strengthened. When POS is lower, which means employees perceive little support from organizations, the impact of GHRM on employees’ OBSE will be weakened. The findings that the interaction of GHRM and POS saliently influence the establishment of OBSE of employees provide meaningful suggestions to managers striving to improve OBSE of employees to realize better performance. This means organizations interested in improving employees’ OBSE should not only implement GHRM but also consider the POS of employees. In addition to highlighting the impact of POS on OBSE, this finding is consistent with previous research [51,96,97,98].

### 5.2. Theoretical Implications

The primary contribution is that our study explores and verifies the psychological mechanism of employee green advocacy, filling a gap in green advocacy literature. In order to protect the environment and realize environmental sustainability, green advocacy was proposed decades ago to persuade people to behave in an environmentally friendly manner [4,7,25]. The majority of research concerning green advocacy focused on ecologists [13,99], social groups, and non-profit organizations [12,15]. Limited studies paid attention to employee green advocacy, and few of them explored the psychological mechanism of it [17,24,25]. In order to fill this gap, our study introduces OBSE as the psychological mediation functioning in the relationship between GHRM practices and green advocacy, proposing and validating empirically the first psychological mechanism of employee green advocacy.

Second, this study not only enlarges studies of green advocacy, but enriches GHRM literature by exploring the positive relationship among GHRM, OBSE, and employee green advocacy. The results of this study respond to calls from Tang and her colleagues [40], who indicate the great importance of exploring more mediating mechanisms in the relationship between GHRM and its related outcome variables. Moreover, prior research concerning GHRM outcomes focuses mainly on organizational performance [44,100,101] and less on employees’ behaviors and attitudes [102]. This study provides OBSE, a kind of psychological attitude of employees, and employee green advocacy, a specific variety of employee behaviors, as outcomes of GHRM, enriching studies concerning GHRM.

Finally, we enrich the POS literature by examining the moderating role of POS in the relationship between GHRM and OBSE. The salient relevance between POS and OBSE is widely acknowledged by large studies [96,97,98]. However, most of the studies discuss the direct linkage between POS and OBSE [96,97,98]. Little research explores the possible moderating role of POS in influencing OBSE. Our study tries to fill this gap by indicating that POS plays a moderating role in the relationship between GHRM and OBSE.

### 5.3. Practical Implications

To begin with, companies are suggested to attach importance to employee green advocacy. Given the pressure from stakeholders to realize environmental sustainability [59,103], managers could encourage employee green advocacy to improve environmental performance [23,104,105]. Our study indicates that employee green advocacy will flourish when the initiatives of GHRM are implemented and when employees’ OBSE is improved, which provides a new method for managers to bolster employee green advocacy. The results of our study suggest that managers could stimulate employee green advocacy by enacting GHRM and facilitating employees’ OBSE [17,21].

Second, managers are recommended to implement GHRM practices to improve their environmental performance. Specifically, organizations could implement green recruitment and selection by taking employees’ environmental values and capabilities into consideration when recruiting and selecting suitable candidates. Organizations could also put green training into practice, enriching employees’ environmental awareness and cultivating employees’ green knowledge and skills. Setting a transparent green performance management system and evaluating employees’ green performance fairly are also sensible ways for organizations to not only improve employees’ green performance but promote them to become green advocates. Moreover, rewarding employees with monetary and non-monetary benefits for employees’ achievement in realizing green goals (green pay and rewards), and providing opportunities to subordinates to participate in environmental management (green involvement) are also recommended. Above all, these specific GHRM practices will help organizations to fully stimulate the willingness and competence of employees to participate in green advocacy.

Further, organizations should pay attention to employees’ OBSE, their psychological condition. To achieve organizations’ long-term development, it is necessary for managers to care about not only organizations’ performance but also employees’ psychological condition [106,107,108]. This study reminds managers of the significance of OBSE, one of the main psychological factors that makes life meaningful [109], by linking it with employee green advocacy. An advisable way is provided in this study for companies to satisfy employees’ OBSE. Results present that employees’ OBSE can be enhanced by the interaction of GHRM and POS, indicating that managers should value employees’ contributions and care about their well-being in the process of GHRM to reinforce employees’ OBSE.

Finally, organizations are supposed to provide support to employees given the importance of POS. Our study emphasized the significance of POS by exploring the vital role it plays in the relationship between GHRM and OBSE. Managers could take various measures to provide support to employees [46]. For example, in the process of green performance management, one of the practices of GHRM, managers could give reasonable feedback to employees, helping them find obstacles prohibiting them from realizing green goals set in a green performance management system and giving them advice about how to solve those problems. Besides, in the process of green involvement, another practice of GHRM, managers could give employees autonomy to engage in green issues and encourage them to deal with green problems with that autonomy [110]. Such instrumental feedback and empowerment could make employees feel that they are supported by the organization, thus contributing to the outcome of GHRM and the improvement of employees’ OBSE.

### 5.4. Limitations and Future Research

The limitations of our study are reflected in the following aspects. First, this research was implemented in China, whereas most measurement tools of this research (except for the scale of GHRM) were developed in the context of western countries. Although these tools have been proved to be stable in the measurement of either Chinese or western samples, choosing local measurement tools is a better choice for researchers to make the measurement of variables conform more to Chinese local reality. Therefore, we suggest future researchers develop measuring scales of OBSE, POS, and employee green advocacy in the context of China.

Second, based on cognitive consistency theory, this study proposed one potential factor (GHRM) and the psychological mechanism (OBSE) that influence employee green advocacy. However, the antecedents and psychological mechanisms that facilitate employees to engage in green advocacy could be varied. Future research is suggested to explore and test more potential factors and psychological mechanisms that have impacts on employee green advocacy from different perspectives.

Third, all measures in this study are self-reports. We suggest future researchers adopt additional methods, including collecting objective data and organizational manipulations, such as Pierce et al. [29] in their validation work.

## Figures and Tables

**Table 1:** The demographic composition of the respondents.

Demographic Information	Number of Interviewees	Proportion (%)
Age		
20–29	34	25.1
30–39	75	55.5
>40	26	19.4
Education level		
<Undergraduate education	79	58.6
≥Undergraduate education	56	41.4
Tenure		
<3 years	32	23.4
3–5 years	41	30.5
5–10 years	39	29.1
>10 years	23	17
Employment type		
Temporary workers	15	11.4
Formal workers	120	88.6
Organizational size		
<20	6	4.4
20–50	15	11
50–100	53	39.3
100–500	42	31.2
>500	19	14.1

**Table 2:** The results of confirmatory factor analysis.

Model	*χ* ^2^	*df*	*χ*^2^/*df*	CFI	TLI	SRMR	RMSEA
4 Factors ^a^	48.442	38	1.274	0.993	0.989	0.036	0.000, 0.081
3 Factors ^b^	81.761	41	1.994	0.971	0.961	0.055	0.059, 0.115
3 Factors ^c^	81.118	41	1.978	0.972	0.962	0.068	0.058, 0.114
2 Factors ^d^	346.102	43	8.049	0.785	0.725	0.129	0.210, 0.255
1 Factor ^e^	517.858	44	11.770	0.664	0.581	0.164	0.265, 0.309

Notes: n = 135. *χ*^2^ = Chi squared, TLI = Tucker–Lewis index, CFI = comparative fit index, SRMR = standardized root mean square residual, RMSEA = root-mean-square error of approximation. ^a^ GHRM, employee green advocacy, OBSE, and POS load on their respective factors. ^b^ GHRM and OBSE load on their respective factors, and employee green advocacy and POS load on one factor. ^c^ GHRM and employee green advocacy load on their respective factors, and OBSE and POS load on one factor. ^d^ GHRM and employee green advocacy load on one factor, and OBSE and POS load on a second factor. ^e^ All indicators load on one single factor.

**Table 3:** Reliability and validity of the constructs.

Construct	Estimate Factor Loading	Composite Reliability (CR)	Convergence Validity (AVE)	Discriminant Validity			
				GHRM	EGA	OBSE	POS
GHRM	0.905~971	0.971	0.872	***0.934***			
OBSE	0.824~909	0.969	0.755	0.424 **	***0.869***		
EGA	0.848~889	0.907	0.764	0.411 **	0.650 **	***0.874***	
POS	0.761~915	0.913	0.642	0.406 **	0.571 **	0.581 **	***0.801***

Notes: n = 135. GHRM is green human resource management, EGA is employee green advocacy, OBSE is organization-based self-esteem, POS is perceived organizational support. The abbreviation for the constructs applies to later tables and figures. Bold italic is the square root of the AVE and the discriminant validity will be sufficiently established if it is greater than the correlation shared between the target construct and other constructs in the model (Fornell and Larcker 1981); composite reliability (CR) > 0.7 is accepted (Hair 1998); average variance extracted (AVE) > 0.5 is accepted (Fornell and Larcker, 1981); two-tailed; ** *p* < 0.01, two-tailed.

**Table 4:** Means, standard deviation, and correlations between study variables.

Construct	1	2	3	4	5	6	7	8	9
1. GHRM	1								
2. OBSE	0.424 **	1							
3. EGA	0.411 **	0.650 **	1						
4. POS	0.406 **	0.571 **	0.581 **	1					
5. OT	0.311 **	0.529 **	0.468 **	0.277 **	1				
6. OS	0.250 **	0.791 **	0.713 **	0.413 **	0.588 **	1			
7. Age	−0.046	−0.021	−0.056	0.051	−0.060	0.013	1		
8. Education	0.126	0.271 **	0.179 *	−0.018	0.040	0.224 **	−0.300 **	1	
9. ET	−0.122	−0.064	−0.038	0.024	−0.046	−0.058	−0.123	−0.104	1
Mean	4.076	4.0052	4.018	3.557	3.608	3.762	33.560	3.448	1.144
SD	0.594	0.445	0.513	0.384	0.555	0.520	6.214	0.643	0.212

Notes: n = 135. OT is organizational tenure, OS is organizational size, ET is employment type; * *p* < 0.05, two-tailed; ** *p* < 0.01, two-tailed.

**Table 5:** Process macro results for mediation analyses.

Regression Analysis	Coeff	se	*t*	*p*	LLCI	ULCI
Dependent variable = OBSE ^a^						
Predictor variables						
GHRM	0.318	0.057	5.608	0.000	0.206	0.431
Dependent variable = EGA ^a^						
Predictor variables						
GHRM	0.119	0.057	2.073	0.040	0.005	0.232
OBSE	0.563	0.100	5.644	0.000	0.366	0.761
Total effect of GHRM on EGA	Effect	SE	t	*p*	LLCI	ULCI
	0.213	0.061	3.495	0.001	0.093	0.334
Direct effect of GHRM on EGA	coeff	se	t	*p*	LLCI	ULCI
	0.119	0.057	2.073	0.040	0.005	0.232
The indirect effect of GHRM on EGA via	Effect	Boot ^b^ SE	Boot LLCI ^c^		Boot ULCI ^c^	
OBSE	0.095	0.040	0.034		0.193	
Normal theory tests for indirect effect	Effect	se	Z		*p*	
	0.095	0.032	2.916		0.004	

Notes: n = 135. Control variable = age, education, organizational tenure, organizational size, and employment type. ^a^ Direct effect; ^b^ 1000 bootstrap samples used; ^c^ LLCI = lower limit of 95% confidence interval (CI); ULCI = upper limit of 95% CI.

**Table 6:** The moderating effects of perceived organizational support.

Model	R	R-sq	MSE	F	df1	df2	*p*
	0.730	0.532	0.089	17.933	8.000	126.000	0.000
Outcome: OBSE		coeff	se	t	*p*	LLCI	ULCI
POS		0.263	0.086	3.037	0.003	0.092	0.434
GHRM		0.149	0.050	3.003	0.003	0.051	0.247
int_1 GHRM * POS		0.252	0.109	2.319	0.022	0.037	0.467
R-square increase due to interaction(s)		R2-chng	F	df1	df2	*p*	
int_1 GHRM * POS		0.02	5.378	1	126	0.022	
Conditional effect of GHRM on OBSE at values of the moderator(s)		Effect	se	t	*p*	LLCI	ULCI
		0.050	0.057	0.885	0.378	−0.062	0.162
		0.149	0.050	3.003	0.003	0.051	0.247
		0.247	0.073	3.396	0.001	0.103	0.392

Notes: n = 135. Control variable = age, education, organizational tenure, organizational size, and employment type. * *p* < 0.05.

## Data Availability

Some or all data and models that support the findings of this study are available from the corresponding author upon reasonable request.

## Appendix A

We used the measurement scales given below in our questionnaires to measure the constructs of GHRM, OBSE, POS and employee green advocacy. The detailed items of variables of this study are showed below. Unless otherwise specified, all measures in this study were evaluated by a 5-point Likert scale ranging from 1 (strongly disagree) to 5 (strongly agree) as described below.

**Green Human Resources Management (GHRM)**
The company’s green claim makes the company more attractive to candidates who regard environmental protection as their job-hunting standard.
The company uses green employer branding to attract green employees.
The company recruits employees who have green awareness.
The company’s training for employees includes environmental management.
The company has integrated training to create the emotional involvement of employees in environment management.
The company has green knowledge management (link environmental education and knowledge to behaviors to develop preventative solutions).
The company uses green performance indicators in our performance management system and appraisals.
The company sets green targets, goals, and responsibilities for managers and employees.
In the company, managers have set objectives on achieving green outcomes included in appraisals.
In the company, there are dis-benefits in the performance management system for non-compliance or not meeting environment management goals.
The company makes green benefits (transport/travel) available rather than giving out pre-paid cards to purchase green products.
In the company, there are financial or tax incentives (bicycle loans, use of less polluting cars).
The company has recognition-based rewards in environment management for staff (public recognition, awards, paid vacations, time off, gift certificates).
The company has a clear developmental vision to guide the employees’ actions in environment management.
In the company, there is a mutual learning climate among employees for green behavior and awareness in my company.
In the company, there are a number of formal or informal communication channels to spread green culture in our company.
In the company, employees are involved in quality improvement and problem-solving on green issues.
The company offers practices for employees to participate in environment management, such as newsletters, suggestion schemes, problem-solving groups, low-carbon champions and green action teams.
**Employee green advocacy**
I tried to persuade team members to reuse and recycle office supplies in the workplace.
My colleagues and I make joint effort to create a more environmentally friendly working environment.
I share with my colleagues the knowledge, information, and suggestions on preventing pollution in the workplace.
**Organization-based self-esteem (OBSE)**
I count around here.
I am taken seriously.
I am important.
I am trusted in my work.
There is faith in me.
I can make a difference.
I am valuable.
I am helpful.
I am efficient.
I am cooperative.
**Perceived organizational support (POS)**
The organization values my contribution to its well-being.
The organization really cares about my well-being.
The organization considers my goals and values.
Help is available from the organization when I have a problem.
The organization can forgive me an honest mistake.
The organization is willing to help me when I need a special favor.
